# Active metal–graphene hybrid terahertz surface plasmon polaritons

**DOI:** 10.1515/nanoph-2022-0189

**Published:** 2022-06-20

**Authors:** Mingming Feng, Baoqing Zhang, Haotian Ling, Zihao Zhang, Yiming Wang, Yilin Wang, Xijian Zhang, Pingrang Hua, Qingpu Wang, Aimin Song, Yifei Zhang

**Affiliations:** Shandong Technology Center of Nanodevices and Integration, School of Microelectronics, Shandong University, Jinan, Shandong, China; Department of Opto-electronics and Information Engineering, School of Precision Instruments and Opto-electronics Engineering, Tianjin University, Tianjin, China; School of Electrical and Electronic Engineering, University of Manchester, Manchester, M13 9PL, United Kingdom

**Keywords:** frequency modulation, graphene, phase modulation, surface plasmon polariton, terahertz

## Abstract

Surface plasmon polaritons (SPPs) are propagating electromagnetic surface waves with local electric field enhancement and nondiffraction limit at optical frequencies. At terahertz (THz) frequencies, a metal line with periodic grooves can mimic the optical SPPs with the same high cut-off response, which is referred to as designer SPPs. Here, by replacing metal grooves with graphene sheets, a novel active metal–graphene hybrid SPP device achieves significant phase modulation. Theoretically, the dispersion curves of THz SPPs are determined by the dimensions and periodicity of the grooves. Changing the chemical potential of graphene sweeps the effective groove depth, which correspondingly shifts the SPP cut-off frequency and modulates the slow-wave phase. A prototype device is fabricated and characterized under varying bias applied for graphene. The experiment demonstrates that the cut-off frequency red shifts from 200 to 177 GHz, and the phase variation is as large as 112° at 195 GHz under a low bias from −0.5 to 0.5 V. Simultaneously, the SPP transmittance is modulated by a factor of more than 3 dB from 140 to 177 GHz due to the graphene absorption. The proposed structure reveals a novel approach to study the nonreciprocal and topological SPPs with active modulation in the THz range.

## 1 Introduction

Surface plasmon polaritons (SPPs) are electromagnetic surface waves propagating along the interface of metal and dielectric with opposite dielectric constants, which exhibit fabulous features of nondiffraction limit and strong electric field confinement [1, 2]. They have found many attractive applications [3, 4] such as highly sensitive biosensing [5, 6], super-resolution imaging [7, 8], ultrahigh-efficiency photovoltaic [9], etc. In physical terms, SPPs are dispersive slow-waves, whose dispersive curves gradually deviate from the light line and eventually approach an asymptotic cut-off limit with enhanced slow-wave properties [2, 4]. Originally, SPPs were reported in the visible and infrared regimes, where the noble metals with plasmonic properties show negative permittivity [10, 11]. At terahertz (THz) frequencies, where the noble metals are typically considered as perfect electric conductors (PECs), structured PECs with sub-wavelength periodic units can mimic the optical SPPs with similar nondiffraction limit and local field enhancement [12, 13]. These designed SPPs address low propagation loss, weak mutual coupling, and simple integration with traditional transmission lines [11, 14, 15], which enables many promising THz applications, such as the 6th generation communication and phased array antenna [3]. Differing from the canonical optical SPPs, the dispersion properties of THz SPPs are typically tailorable by changing the dimensions of the periodic structures [13, 15, 16].

Typically, the metallic SPP structures have fixed optical properties after design, which can be dynamically modulated by combining with active stimuli, such as semiconductor diodes [17, 18], ferroelectric materials [19], temperature-sensitive materials [20]. Varactor diodes with tunable capacitance have been investigated to sweep the resonant frequencies of small resonators [17], and Schottky diode with tunable resistance can change the resonant magnitude [18]. On the other hand, active frequency modulation has been investigated with barium strontium titanate (BST) [19], and broadband amplitude modulated has been reported with VO_2_ [20]. In addition to these relatively bulky materials and devices, graphene, i.e., a single layer of carbon atoms, is a promising modulation stimulus with unique properties of high electron mobility, good optical transparency, excellent thermal conductivity, and tunable electrical conductivity under electric field [21–23]. Broadband amplitude modulation of SPPs have been demonstrated by using graphene at both microwave and optical frequencies. However, graphene-based phase and frequency modulation have not been reported for designer SPPs. To the best of the authors’ knowledge, active phase modulation has not been reported for THz SPPs yet, which is of great importance in no-reciprocal and phase-delay devices.

In this paper, we propose an active metal–graphene hybrid SPP device with significant phase modulation at THz frequencies for the first time. The SPP structure consists of periodic graphene grooves on a metal line as a substitution of the conventional metallic grooves, whose effective depth can be tuned by applying gate bias. In this case, the SPP cut-off frequency red shifts as the effective depth increases, and thus the slow-wave phase is significantly modulated. To apply uniform gate field for graphene, periodic metallic tips are designed on the graphene grooves, which slightly changes the initial cut-off frequency. A prototype with co-planar waveguide (CPW) feedings is fabricated and characterized. The measured cut-off frequency is modulated from 200 to 177 GHz under a low bias voltage of less than 1 V, which corresponds to a phase modulation of 112° at 195 GHz. Simultaneously, the transmittance modulation is as large as 3 and 7 dB at 140 and 170 GHz, respectively.

## 2 Methods and experiments

### 2.1 Eigen mode analysis

**Figure 1:**
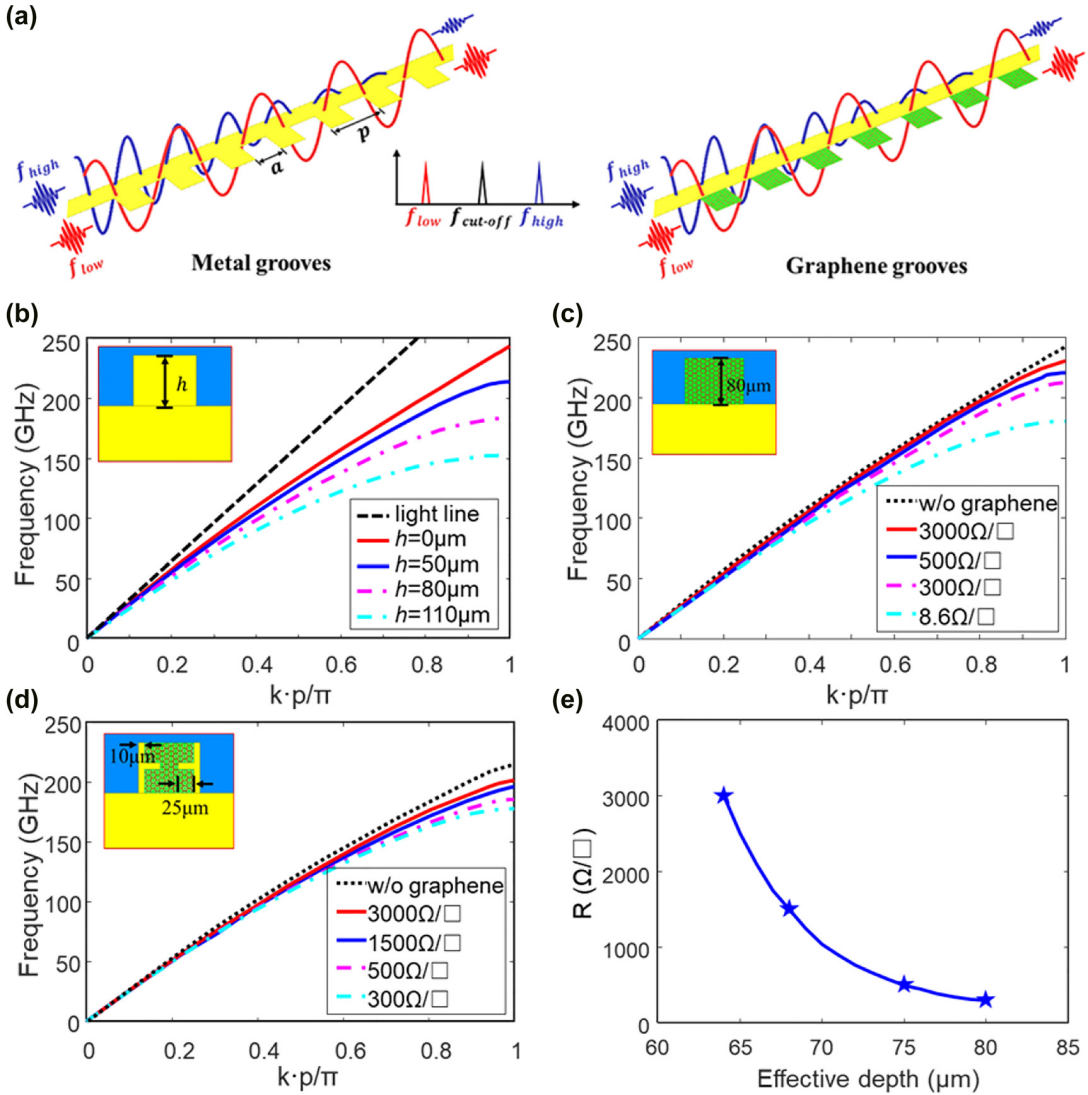
THz SPPs and their dispersion curves. (a) Metal SPP and metal–graphene hybrid SPP structures (*p* = 0.21 mm, *a* = 0.1 mm). Dispersion curves of (b) metal grooves with various depth, (c) graphene grooves at various sheet resistances for graphene, and (d) graphene grooves with metal tips at various sheet resistances for graphene (*h* = 80 μm). The insets show the unit cell of THz SPPs. (e) Correspondence of resistance and effective depth of graphene grooves.

Typically, a metal line with periodic metallic grooves guides THz SPPs in Figure 1(a), whose propagation constant is given by

(1) $$\beta =\sqrt{\varepsilon_{\text{eff}}k_{0}^{2}+{\left(a/p\right)}^{2}\varepsilon_{\text{eff}}k_{0}^{2}\tan^{2}\left(k_{0}\sqrt{\varepsilon_{\text{eff}}}h\right)}$$

where *a* is the width of the groove, *p* is the lattice period, *h* is the groove depth, *k*_0_ is the wave number of the light in free space, and *ɛ*_eff_ is the effective permittivity for metal grooves [24]. It can be seen that THz SPPs are dispersive, whose dispersion relation is leveled at a cut-off frequency

(2) $$\omega_{p}=\pi c_{0}/2\sqrt{\varepsilon_{\text{eff}}}h$$

where *c*_0_ represents the free space light speed [24]. Sweeping the groove depth *h* changes the cut-off frequency of THz SPPs.

To precisely predict this trend, the dispersion curves of THz SPPs are simulated using Eigen Mode Solver in Ansys High Frequency Structural Simulator (HFSS). Figure 1(b) depicts one unit of a classic SPP structure and its dispersion curves with varying groove depth and fixed groove width and lattice period. The cut-off frequency decreases as the groove depth *h* increases, showing good match with Eqs. (1) and (2). Considering the spectral range of our measurement system, *h* = 80 μm is chosen for the following analysis, which corresponds to a cut-off frequency of 180 GHz.

To actively change the groove depth, the metal grooves are substituted with graphene grooves, as shown in the inset of Figure 1(c). The effective depth of these graphene grooves can be estimated with an attenuation length ζ, i.e., the distance where the electromagnetic waves attenuate to 1/*e* [25]. At terahertz frequencies,

(3) $$\zeta =-\frac{{\left|\sigma_{g}\right|}^{2}}{2\omega \varepsilon_{d}\sigma_{g}^{\prime \prime }},\sigma_{g}^{\prime \prime }<0$$

where *ω* is the angular frequency, *ɛ*_d_ is the relative permittivity of the dielectric, and $\sigma_{g}^{\prime \prime }$ is the imaginary part of *σ*_g_ [25]. *σ*_g_ is typically determined by the intraband term of the Kubo formula at THz frequencies, and the sheet resistance of graphene is estimated as from 500 to 8.6 Ω/□ at a phenomenological scattering rate of 2 × 10^12^ s^−1^ [26]. The simulated dispersion curves are illustrated in Figure 1(c), where the cut-off frequency red shifts from 220 to 180 GHz. However, the transferred chemical vapor deposition (CVD) graphene typically has much larger resistance than the ideal numbers due to the defects and chemical residues [27]. In this regard, we modified the simulation with a resistance sweeping from 3000 to 300 Ω/□, which is extracted from the direct-current (DC) data of a graphene field effect transistor, see the Supplementary Information. As the graphene resistance decreases from 3000 to 300 Ω/□, the cut-off frequency shifts merely from 232 to 213 GHz.

To apply uniform electric field on graphene, periodic metallic tips are designed on the metal line, which slightly changes the dispersion curve, see the black dotted line in Figure 1(d). As the resistance reduces from 3000 to 300 Ω/□, the cut-off frequency sweeps from 204 to 180 GHz, showing much larger modulation range. The corresponding effective depth of the graphene grooves is estimated as from 64 to 80 μm by fitting the metallic grooves, as shown in Figure 1(f).

### 2.2 Full wave simulation

**Figure 2:**
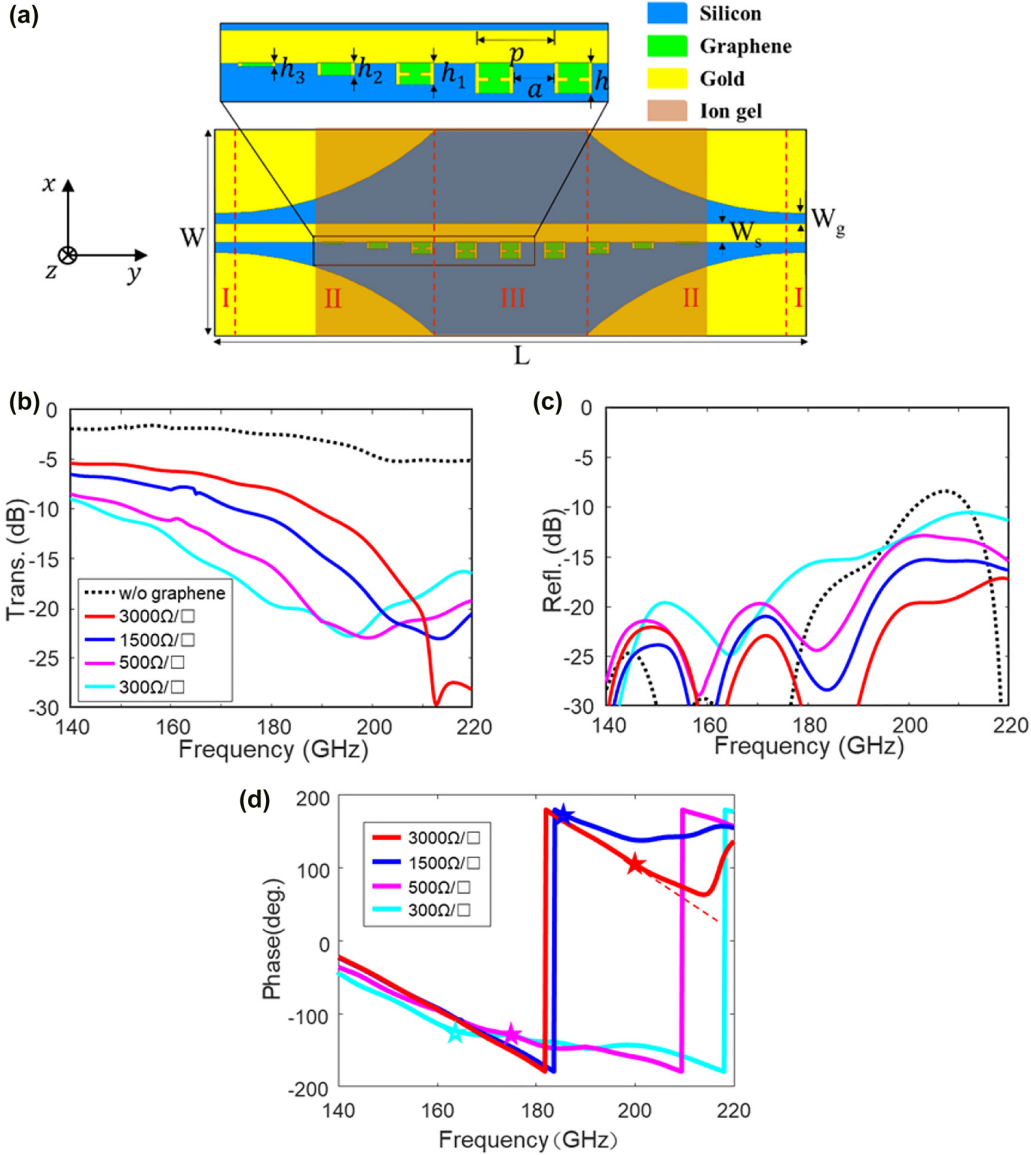
Simulation of a metal–graphene hybrid structure with CPW feeding. (a) 3-D model (*h*_1_ = 58 μm, *h*_2_ = 34 μm, *h*_3_ = 10 μm, *W* = 1.5 mm, *L* = 2.8 mm, *W*_s_ = 87 μm, *W*_g_ = 5 μm). Simulated (b) transmittance, (c) reflectance, (d) transmittance phase at various sheet resistances. The star marker depicts the phase deviation point with respect to linear phase.

**Figure 3:**
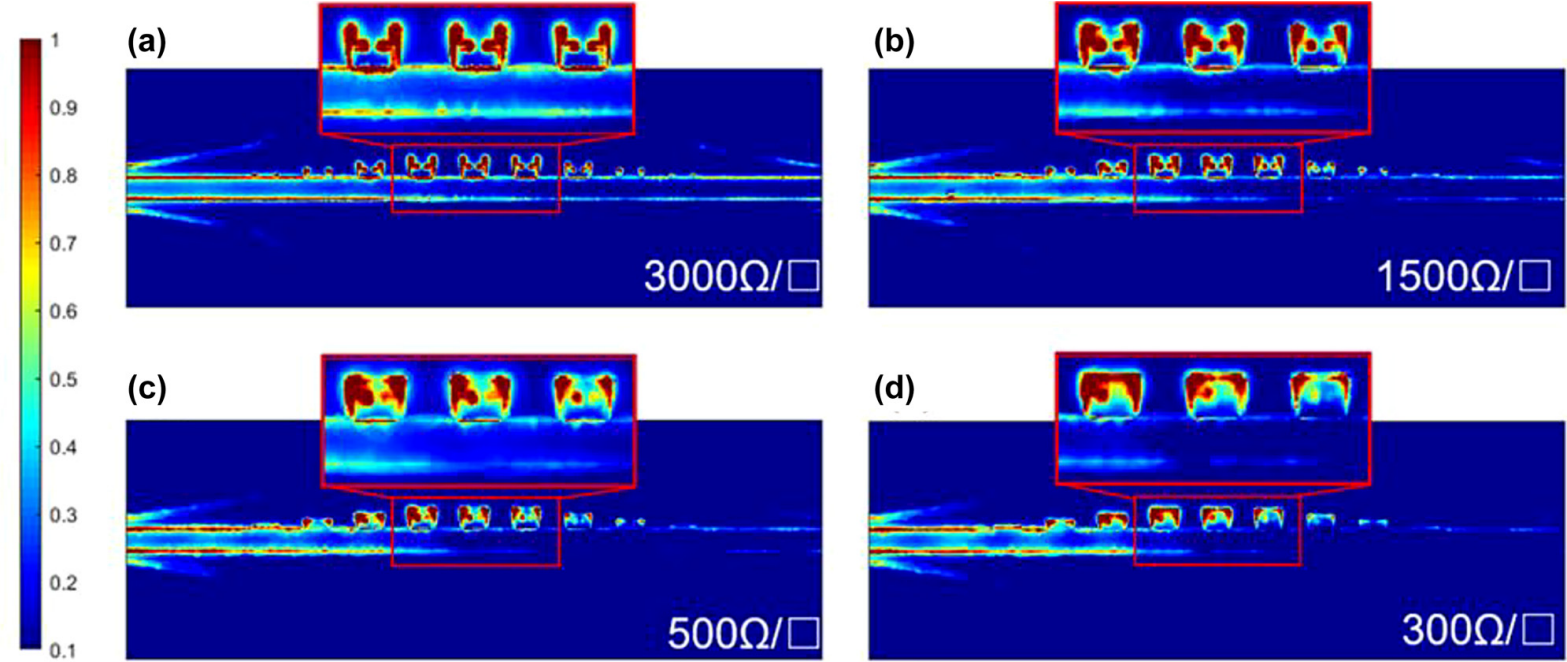
Electric field distributions of the hybrid structure at various graphene resistance. (a) 3000 Ω/□, (b) 1500 Ω/□, (c) 500 Ω/□, and (d) 300 Ω/□. The concentrated fields move towards the outer edge of graphene grooves as the resistance decreases.

Figure 2(a) illustrates a metal–graphene hybrid model on a 200-μm thick silicon substrate designed in Driven Mode Solver of HFSS, consisting of two 50-Ω CPWs (I), two CPW-SPP transitions (II), and periodic graphene grooves and bias tips (III). The design principles of the CPWs and transitions are not the contribution of this work, and thus not discussed here, which can be found in our previous paper [18]. Their optimized dimensions are listed in the caption of Figure 2. Considering graphene biasing, a thin-layer styrenesulfonic acid sodium salt (PSSNa) is designed on the surface of the proposed device, whose relative permittivity is around 3 [28, 29]. The simulated transmittance and reflectance are illustrated in Figure 2(b) and (c), respectively. The metal structure without graphene is simulated as a reference, which has an insertion loss of less than 2.5 dB below 180 GHz. As the graphene resistance decreases, the cut-off frequency of the hybrid device red shifts, and the SPP attenuation enlarges due to the enhanced graphene absorption. Considering the fabrication deviations, tolerance analysis has been investigated and reveals little impact on the transmittances, as shown in Figure 2 of Supplementary Information. This phenomenon can be explained by the electric field distributions in Figure 3. As the graphene resistance decreases from 3000 to 300 Ω/□, the concentrated electric fields move towards the graphene edges, which reveal that the effective depth of the graphene grooves increases. Consequently, the cut-off frequency red shifts and the graphene absorption enhances. The SPP transmittance is modulated from −5.4 to −9 dB at 140 GHz and from −8 to −19.5 dB at 180 GHz. However, it is not easy to tell the cut-off frequency precisely due to the graphene absorption, as shown in Figure 2(b) and (c). To address this issue, the phase of the transmittance is plotted in Figure 2(d). As the frequency approaches the cut-off frequency, the slow-wave effect induces enhanced phase delay. As long as the phase deviates from the linear curves, the cut-off frequency is very close. Take 3000 Ω/□ for instance, the phase deviation occurs at 200 GHz, and the cut-off frequency is at 204 GHz.

### 2.3 Fabrication and measurement

**Figure 4:**
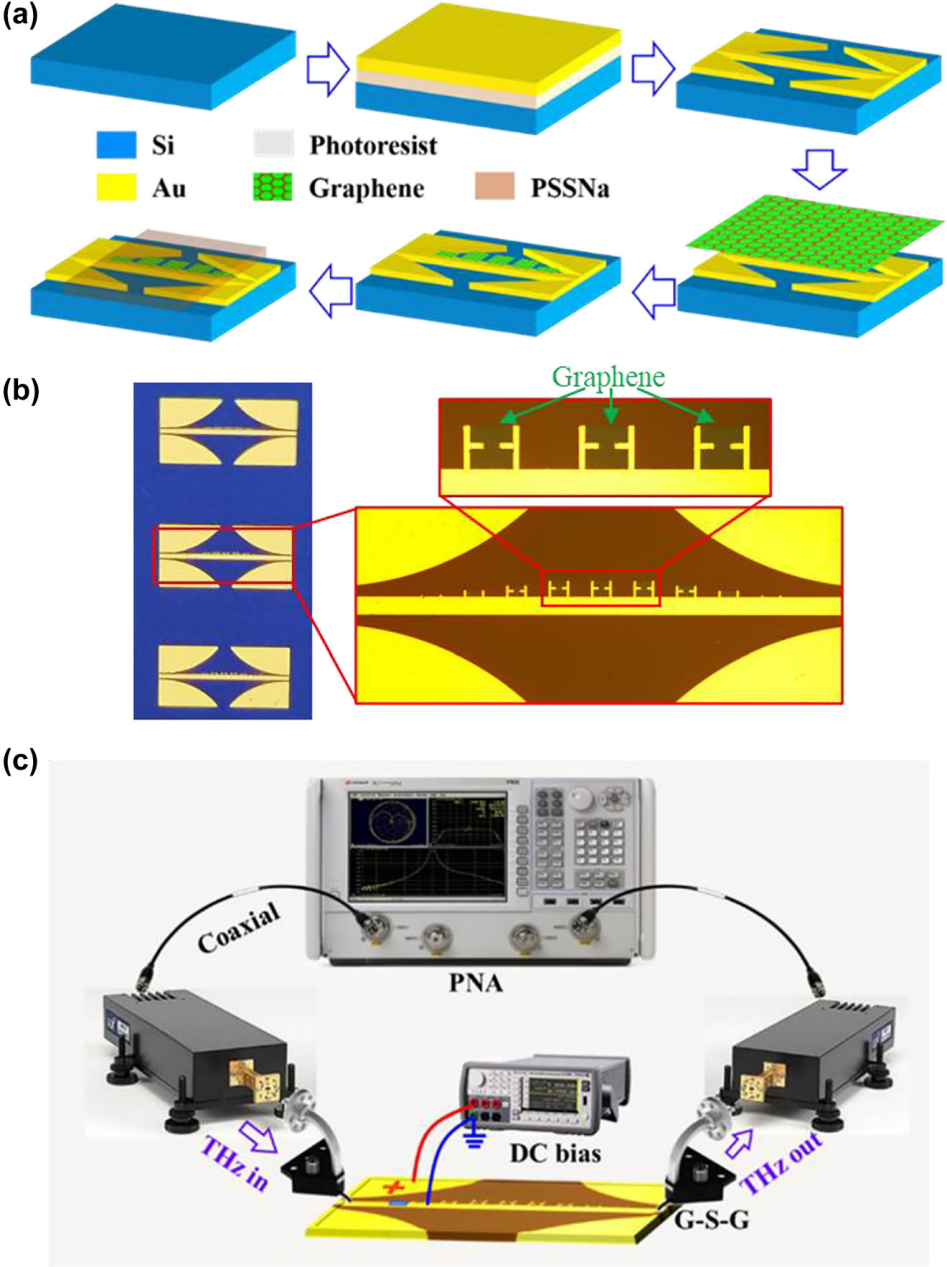
Fabricated metal–graphene hybrid SPP structure. (a) Fabrication steps, (b) image of the fabricated device, and (c) measurement system set-up.

The proposed device was fabricated following the steps in Figure 4(a). Firstly, metal structure patterns were defined on a 200-μm thick silicon substrate with a 100-nm oxide layer by using standard ultraviolet photolithography. 5-nm Ti and 200-nm Au films were deposited with e-beam evaporation, and then the circuit patterns were obtained by using lift-off with acetone. Next, a CVD graphene film was transferred onto the device and patterned with photolithography and dry etching. The details of wet transfer process can be found in Supplementary Information, and the patterned graphene is designed to have a 5-μm overlap region with the metal lines and tips to ensure good contact. The fabricated device is illustrated in Figure 4(b). Then, a 70-μm thick layer of solution-processed PSSNa was spin-coated onto the device, which forms an electric double-layer capacitor (EDLC) for gating graphene. Figure 4(c) illustrates the measurement set-up. Two 50-Ω ground–signal–ground (G–S–G) probes integrated with Agilent programmable network analyzer (PNA) were employed to characterize the transmittance and reflectance of the device, which were calibrated with short-load-open-through (SLOT) method and then launched on the two CPWs of the device for feeding and receiving signals. During the spectral test, the DC bias was applied on the EDLC between the CPW ground and signal electrodes to sweep the graphene conductivity through a bias-Tee of the PNA, and the spectral variation was recorded by PNA. Considering that PSSNa is a water-based ion gel, the typical DC bias in the experiment should be less than 1.5 V to avoid the electrochemical reaction, which may lead to irreversible damage to the PSSNa EDLC and thus degraded modulation range for graphene conductivity.

## 3 Results and discussion

**Figure 5:**
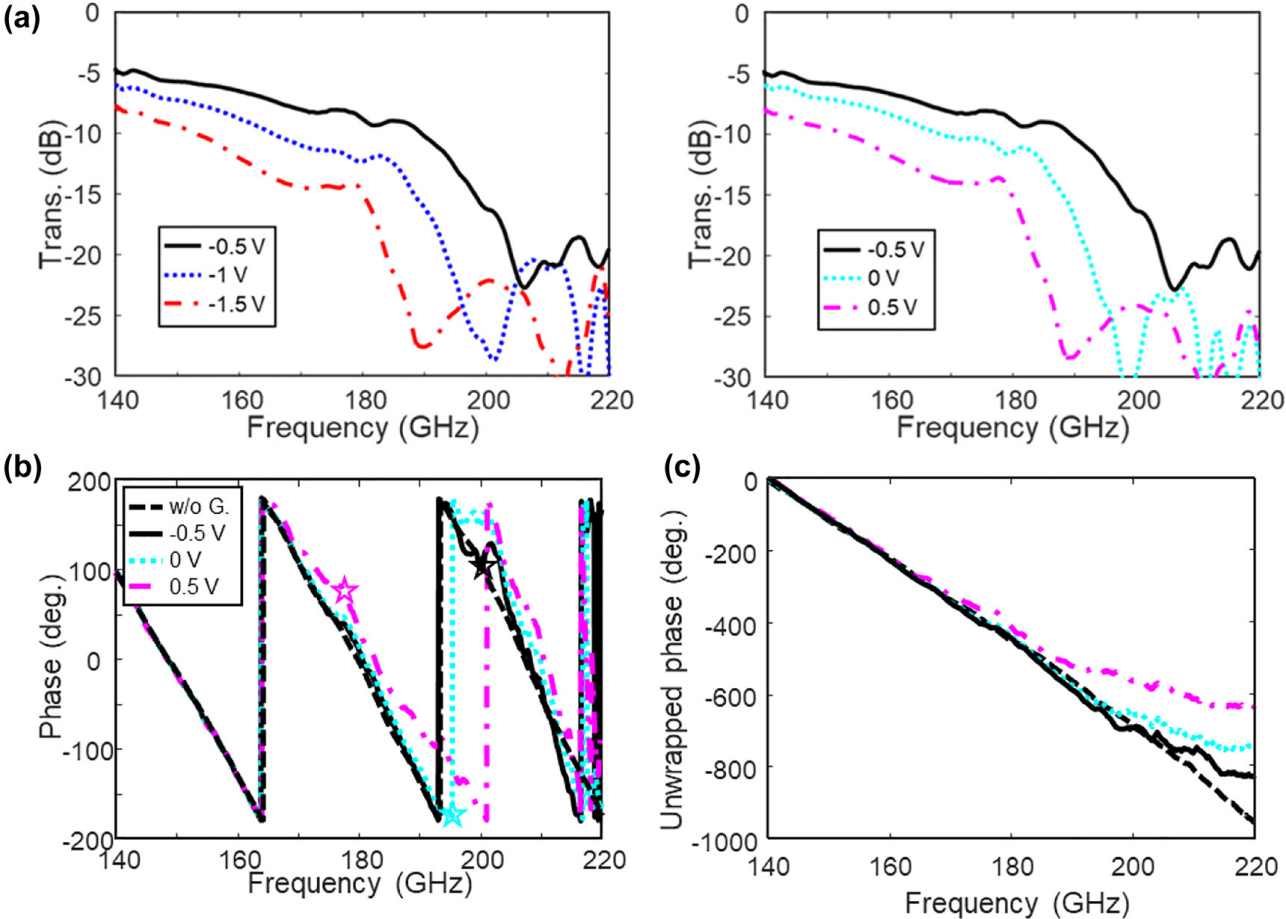
Measured (a) transmittance, (b) wrapped phase, and (c) normalized unwrapped phase under various bias voltages.

The characterized transmittance amplitude and phase under various bias voltages are shown in Figure 5. As can be seen in Figure 1 of the Supplementary Information, the Dirac point of the transferred graphene is approximately at −0.5 V, where the resistance is the maximum. As the applied bias sweeps from −1.5 to −0.5 V, the graphene resistance increases, and the effective groove depth decreases, which blue shifts the cut-off frequency. As the bias further sweeps from −0.5 to 0.5 V, the graphene resistance decreases, and the effective groove depth increases, which red shifts the cut-off frequency. The characterized reflectance curves in Figure 3 of the Supplementary Information illustrate the same shift of the cut-off frequency, whose amplitude is relatively stable. The corresponding phase modulation due to the slow-wave effect is illustrated in Figure 5(b). Under a bias voltage of −0.5 V, a clear phase deviation is observed at 200 GHz, indicating that the cut-off frequency is not far away. As the bias increases to 0.5 V, a phase delay is noticeable at 177 GHz, revealing a red shift of the cut-off frequency and the slow-wave effect. Figure 5(c) illustrates the normalized unwrapped phase, whose variation is as large as 112° at 195 GHz and 180° at 212 GHz under a bias varying from −0.5 to 0.5 V. Simultaneously, the transmittance amplitude is also significantly modulated. As the bias sweeps from −1.5 to −0.5 V, the SPP attenuation decreases from 7.8 to 5 dB at 140 GHz and from 14.5 to 8 dB at 180 GHz due to the weakened graphene absorption; as the bias sweeps from −0.5 to 0.5 V, the attenuation increases from 5 to 8 dB at 140 GHz and from 8 to 15 dB at 180 GHz due to the enhanced absorption. The transmittance variation reveals an on/off ratio of up to 7 dB from 140 to 177 GHz, whose trend versus bias is consistent with the characterized resistance variation in Figure 4 of the Supplementary Information. Here, the applied sub-volt bias in our approach is smaller than the other graphene-modulated active SPP devices [17, 18, 21, 30]. To further enlarge the modulation range, high-quality CVD graphene can be used to provide lager tunable range of graphene conductivity, and graphene transfer techniques may be optimized to minimize the influence of residuals. Furthermore, water-free ion gels with larger capacitance and larger working bias voltages also contribute to graphene modulation range.

## 4 Conclusions

In summary, a novel metal–graphene hybrid THz SPP device was proposed with significant phase modulation by sweeping the SPP cut-off frequency. The hybrid structure comprises periodic graphene grooves on a metal line, whose effective depth is actively controlled with gate bias. Periodic bias tips are designed on the metal line to provide larger modulation range and more uniform gate field for graphene. As the bias voltage varies from −0.5 to 0.5 V, the SPP cut-off frequency red shifts from 200 to 177 GHz due to the increasing effective depth and, more notably, significant phase modulation is observed due to the enhanced slow-wave effect. The phase variation is as large as 112° at 195 GHz, and the transmittance modulation is larger than 3 dB from 140 to 177 GHz due to the enhanced graphene absorption. This work provides a new method to modulate the phase and cut-off frequency of THz SPPs at silicon complementary metal–oxide–semiconductor compatible voltages, which may find applications in no-reciprocal and topological SPP devices as well as beam control of SPP antennas with active phase delay.

## Supplementary Material

Supplementary Material Details

Supplementary Material Details
