# Supplementary material for: Active metal–graphene hybrid terahertz surface plasmon polaritons
Source: Nanophotonics. 2022 Jun 20;11(14):3331–8. doi: 10.1515/nanoph-2022-0189 (PMC11501757; doi:10.1515/nanoph-2022-0189)
Supplement: Supplementary file 1 — Supplementary Material Details [file j_nanoph-2022-0189_suppl_001.docx]

Mingming Feng, Baoqing Zhang, Haotian Ling, Zihao Zhang, Yiming Wang, Yilin Wang, Xijian Zhang, Pingrang Hua, Qingpu Wang, and Aimin Song*, Yifei Zhang*

Active Metal-Graphene Hybrid Terahertz Surface Plasmon Polaritons

**(Supplementary Information)**

1. Conductivity of graphene


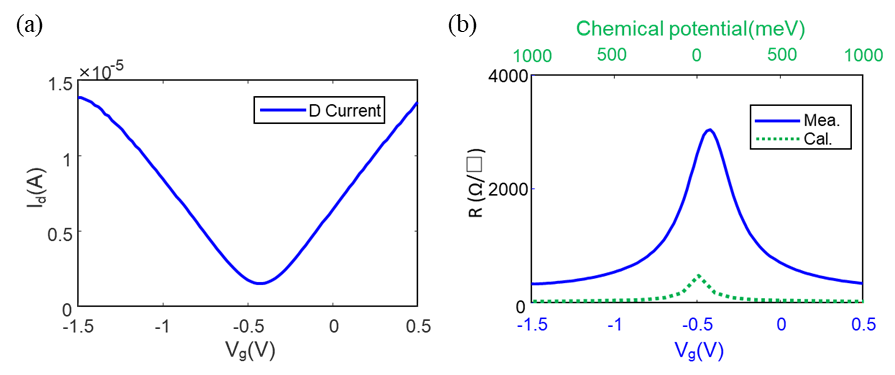


Fig. S-1: (a) I-V response of a graphene transistor using PSSNa as the top gate. (b) Comparison of the sheet resistances extracted from the I-V response and calculated by the Kubo formula.

The graphene transistors are fabricated on the same substrate as the metal-graphene hybrid SPPs device, whose channel length and width are 300 and 350 μm, respectively. The calculated sheet resistance for graphene sweeps approximately from 3000 to 300 Ω/□. Compared to the results calculated by the Kubo formula, the resistivity of CVD graphene is significantly greater.

1. Tolerance Analysis


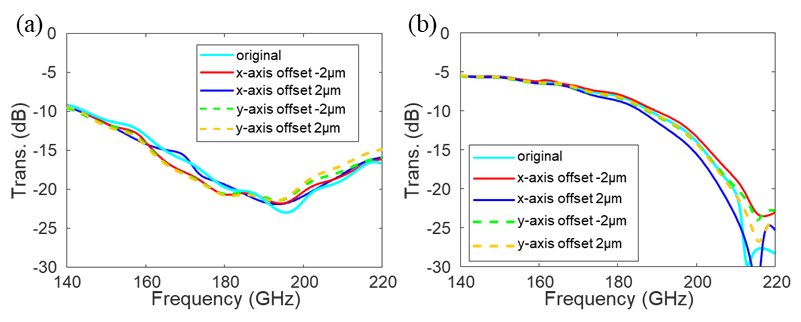


Fig. S-2: Tolerance analysis of fabrication deviations. Simulated transmittances of the device with graphene sheets offset by 2 μm along the x or y axes at a resistance of (a) 300 Ω/□ and (b) 3000 Ω/□.

Simulation results show little variations in the transmittance curves, when the graphene sheets offset by 2 μm.

1. Measured data of the fabricated sample


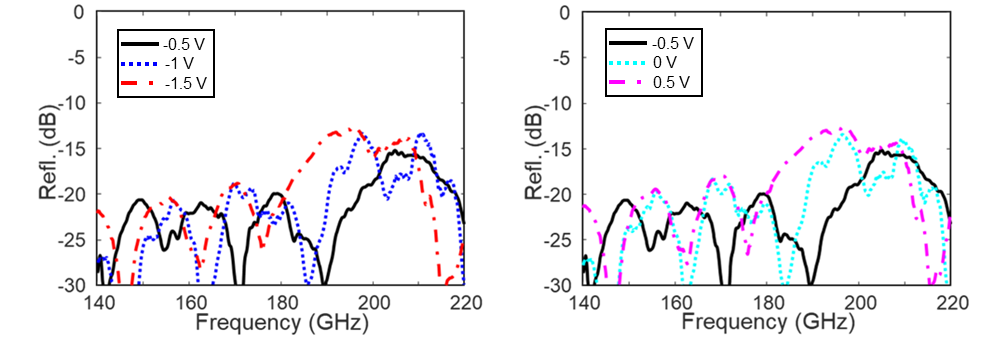


Fig. S-3: Measured reflectance of the fabricated sample under various bias voltages.

The magnitude of reflectance is relatively stable and the sweep of cut-off frequency can also be observed.


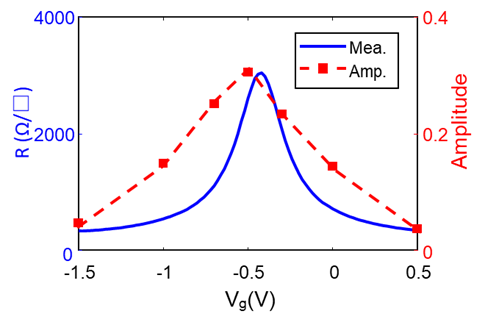


Fig. S-4: Transmission variation of fabricated sample with applied voltage at 190 GHz versus graphene DC characteristics.

At 190 GHz, the transmission is modulated from 0.31 to 0.04 as the bias voltage sweeps from -1.5 to 0.5 V, which can be attributed to the shift of cut-off frequency and the variable intensity of absorption. As the graphene resistance increases, the effective depth becomes smaller, and the transmission enhances, which implies a blue shift of the cut-off frequency; as the resistance decreases, the effective groove depth enlarges, which corresponds to a red shift of the cut-off frequency as well as the decrease of the transmission. The measured DC variation of graphene and hybrid structure show consistent trend, revealing that the active modulation of THz SPPs is contributed to graphene.

1. Details of graphene transfer and biasing

Graphene transfer: The graphene used in this work is CVD graphene grown on copper. First, a layer of polymethyl methacrylate (PMMA) was spin-coated onto the graphene as a supporting layer with a speed of 3000 rpm for 30 seconds. To better protect graphene layer, the spin-coated film was dried on a hotplate at 60°C for 10 minutes. FeCl_3_ solution was used to etch the copper layer without damaging the graphene and PMMA. After removing the copper, the graphene layer with PMMA was clean with de-ionized water to remove the residuals. Then the graphene with PMMA was transferred onto the target substrate, and dried for at least 2 hours. Finally, the PMMA was removed by using acetone, leaving the graphene on the substrate.

PSSNa preparation: PSSNa powder, D-sorbitol, glycerol and de-ionized water were mixed with a weight ratio of 40, 10, 10, and 40%. After ultrasonic and magnetic stirring alternatively for 4 hours at room temperature, quasi-transparent ion gel was obtained. Then ion gel was spin-coated onto the device with a spinning speed of 1000 rpm for 60 seconds without any post annealing step.
